# Embodied Perception: A Proposal to Reconcile Affordance and Spatial Perception

**DOI:** 10.1068/i0709jc

**Published:** 2015-04-01

**Authors:** Rouwen Cañal-Bruland, John van der Kamp

**Affiliations:** Faculty of Human Movement Sciences, MOVE Research Institute Amsterdam, VU University, Amsterdam, The Netherlands; Faculty of Human Movement Sciences, MOVE Research Institute Amsterdam, VU University, Amsterdam, The Netherlands and Institute for Human Performance, The University of Hong Kong, Hong Kong SAR, China

**Keywords:** action, affordance, embodied perception, spatial perception

## Abstract

Proffitt's embodied approach to perception is deeply indebted to Gibson's ecological approach to visual perception, in particular the idea that the primary objects of perception are affordances or what the environment offers for action. Yet, rather than directly addressing affordance perception, most of the empirical work evaluating Proffitt's approach focuses on the perception of spatial properties of the environment. We propose that theoretical and empirical efforts should be directed toward an understanding of the relationship between affordance perception and spatial perception, keeping in mind that this relationship is nontrivial because affordance perception is dichotomous, whereas the perception of spatial properties is gradual. We argue that the perception of spatial properties of the environment is enslaved by affordance perception, most notably at the critical boundaries for action. To empirically scrutinize this proposition, and to solve issues raised regarding the validity of several empirical findings, we call for joint research efforts to further understanding of embodied perception.

Imagine yourself on a hiking trip. You arrive at the bottom of a hill, look at its steep slant and feel your weary-legs. Do you perceive the hill as climbable, or not? Proffitt's theory of embodied perception argues that the visual perception of what the hill offers for action is grounded in the potential costs of the action relative to the current action capabilities (Proffitt, 2006). If costs are high—for example, because you are fatigued and hence are more likely to stumble and fall when climbing the hill—, then the hill appears steeper than it actually is, thereby promoting the choice for an economic—and safe—action (e.g., Bhalla & Proffitt, 1999; Proffitt, 2006; Proffitt, Bhalla, Gossweiler, & Midgett, 1995).

Firestone (2013, p. 455) recently countered that Proffitt's “account of spatial perception not only isn't true—it couldn't be true, even if its empirical findings were accepted at face value,” thereby rejecting Proffitt's (2006, Proffitt & Linkenauger, 2013) embodied approach to perception on both theoretical and empirical grounds. In particular, Firestone (2013) argued that i) the size of the perceptual biases do not relate to the change in action ability of the observers, ii) action-specific units are incommensurate, iii) Proffitt's account misses an informational basis, iv) the perceptual biases are not subjectively noticeable, while they should be, and finally v) the perceptual effects are biases in judgment and not perception. Although we are somewhat equivocal regarding its empirical support, we do agree that there are indeed theoretical issues with Proffitt's approach, but for different reasons than those raised by Firestone. The aim of this comment is to first clarify what we think the pertinent theoretical issues are, to then spark new directions for a theory of embodied perception.

*Step 1: A salute to affordance perception!* Proffitt repeatedly affirms that his embodied approach to perception originates in Gibson's ecological approach to visual perception, particularly the idea of affordances: “I view the current approach as being a development of this idea, and I am deeply indebted to it” (Proffitt, 2006, p. 120). A fundamental proposal in Gibson's functional approach is that perception is for action, and action is for perception. Accordingly, the *primary* objects of perception are affordances, or the opportunities for action that emerge from the correspondence between the observer's action capabilities (constrained by but not determined by body morphology and physiology) and the properties of the environment. It is therefore impossible to think of affordances without affirming the inseparable unity or “complementarity of the animal and the environment” (Gibson, 1986/1979, p. 127). This implicates that perceiving affordances of the environment should not be confused with the perception of a spatial world such as described by physics in terms of metric units of space and time, and which conceives the world as is independent of the observer (Gibson, 1986/1979). Of course, we can perceive spatial properties like distance, size, and slope in meters and degrees, but this is a different kind of perception. Perception is, first and foremost, a matter of directly discovering meanings for action, and not a matter of affixing meaning to physical objects with certain properties (Ingold, 2011).

*Step 2: Affordance perception is not (always) accurately reflected in measures of spatial perception!* Proffitt argues that perception functions to “promote effective actions in the immediate environment” (Proffitt & Linkenauger, 2013, p. 171), thereby firmly rooting his approach in the idea that affordances are the primary objects of perception. However, an embodied approach that is truly grounded in affordance perception demands affordances and associated (realized or not realized) actions to be a, if not *the*, decisive dependent measure. We note, however, that the empirical approach taken by Proffitt and other researchers (including ourselves!) has been dominated by asking observers to make various types of perceptual estimates of the spatial properties of the environment, including distances, sizes, slopes, and speeds, instead of asking participants about affordances or to act upon them (for exceptions, see Lessard, Linkenauger, & Proffitt, 2009; Taylor, Witt, & Sugovic, 2011). This lopsided prevalence for measures of spatial perception is a major problem in the current empirical efforts that address Proffitt's embodied approach to perception. The observers' perception of affordances, the fundamental pillar within the embodied approach to perception, is simply presumed without being empirically delineated. Consequently, we cannot even be sure that the reported distortions in the perception of environmental properties are related to affordance perception, and hence, whether they are truly grounded in action. In other words—as yet—we cannot establish whether or not the reported distortions are indeed indicative of perception being embodied. To solve this question requires measurements of affordance perception—or better acting on affordances.

*Step 3: Affordance perception is dichotomous, spatial perception continuous!* How do spatial and affordance perception differ? According to Gibson's ecological approach, affordances bolster the functional relationship between animal and environment. It follows that affordance perception must be veridical, otherwise perception would not be adaptive and animals (including humans) could not survive. Affordance perception is veridical in the sense that it accurately specifies what an observer currently can or cannot do—usually after a process of adaptation, learning, or development. However, the perception of environmental properties such as size, distance, and slope seems more fallible in the sense that systematic distortions occur relative to the actual measures of the physical environment. Together, this suggests that the perception of affordances and the perception of environmental properties do not map one-to-one, that is, they do not relate in a linear manner. To specify: affordance perception is dichotomous, it defines the boundaries for action. Referring back to our example in the Introduction, a hiker at the bottom of a hill has—in the end—two action options: “Fes, I can/do climb this hill,” or, *“No*, I cannot/do not climb it.” This is not to say that with repetitive encounters, the boundaries for action remain unchanged. For a hiker who slowly fatigues, the boundaries for action will gradually shift, such that a hill that first was climbable, now is not. The typical probabilistic function when plotting affordance perception as a function of environmental properties reflects these dynamic rather than fixed action boundaries across encounters (cf. Franchak & Adolph, 2014; Wagman & Malek, 2009). Yet, for each single encounter, affordance perception is dichotomous. This dichotomy implies that small changes in the environment and/or the observer can abruptly induce a discontinuous change in the perception of what the environment offers for action. By contrast, the perception of the physical properties in the environment is gradual: This hill is shallow, that hill is steep, and that one is very steep (or expressed in angles). Consequently, a small change in the environment evokes a concomitant proportional change in the perception of that property.

*Step 4: A proposal for explaining the distortions in spatial perception.* If we accept the empirical findings at face value, then the reported distortions in the perception of spatial properties of the environment due to manipulations of bodily states call for an explanation. We suggest that this explanation needs to be sought in the relationship between perception of affordances and the perception of spatial properties in the environment. Admittedly, we have no full-fledged account to offer yet, but the following observations may provide some building blocks. We propose that given the functional primacy of affordance perception, it is of utmost importance that its integrity is maintained. If indeed the perception of spatial properties is secondary to or enslaved by affordance perception, then maintaining the integrity of affordance perception may affect (or sacrifice) the accuracy of the perception of the spatial environment, especially for situations that are close (i.e., critical) to the boundaries for action (see also Fajen & Phillips, 2012; Witt & Riley, 2014). This is depicted in Figure 1.

Our reasoning is clearly reminiscent of Proffitt's account and his explanation of the observed perceptual distortions: “This is useful, because the difference between, for example, a 5° hill and a 6° is of considerable importance when planning locomotion, whereas the differences between, say, a 65° cliff and a 66° cliff is of no behavioral significance” (Proffitt, 2006, p. 114). We agree, and argue that the distortions in spatial perception are most pronounced around the action boundaries. In our view, this counters Firestone's critique that Proffitt's embodied approach to perception can only explain the direction of the perceptual error (i.e., over- or underestimations) but not their magnitude. Firestone—mistakenly, we believe—presumes a one-to-one mapping between the perceptions of affordances and environmental properties. Yet, in our reasoning, a backpack twice as heavy should not—as a rule—result in an overestimation of the steepness of the hill that is twice as large. Instead, the magnitude of the perceptual distortion should depend on the correspondence between the action capabilities of the observer and the properties of the environment. In particular, it should be largest close to critical boundaries for action and negligible within and beyond these boundaries. We hypothesize that the magnitude of the distortion is in fact a function of the proximity to the critical action boundary; an empirical evaluation of this hypothesis has yet to be delivered.

In addition, a crucial future endeavor is to develop an account of *why* and *how* the discontinuous changes in affordance perception close to the critical action boundaries impinge on spatial perception and result in systematic over- or underestimations in the perception of environmental properties. Based on ecological psychology, two sources of perceptual inaccuracies can be identified: the observer attends to a nonspecifying informational variable or the informational variable is inappropriately calibrated to perception. Witt and Riley (2014) recently argued that the primacy of affordances perception over spatial perception perhaps leads to the exploitation of nonspecifying variables for spatial perception. We deem it more likely, however, that the enslaving of spatial perception by affordance perception results in inappropriate scaling of the specifying variable to spatial perception, because unlike attunement, imperfect calibration is typically associated with systematic over- or underestimations of perception (e.g., Cabe & Wagman, 2010; Withagen & Michaels, 2005). It follows that distortions of spatial perception should reflect the quality of the calibration process, a further hypothesis that requires future testing.

**Figure 1.**
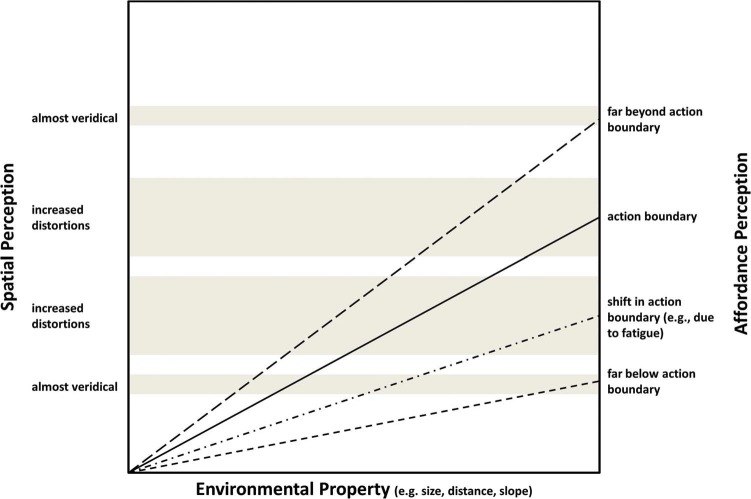
The relationship between the perception of spatial properties and affordances. Close to action boundaries affordance perception distorts spatial perception in a direction consistent with an increased economy of action.

*Step 5: Let's organize a collective effort to systematically replicate the typological studies!* This leads to our final point, namely the discussion about the validity of the empirical findings brought up both in support and in rebuttal of Proffitt's embodied approach to perception. Every researcher involved will agree that the experimental data are decisive in evaluating whether Proffitt's account is correct or not, or more pertinently, why spatial perception is sometimes distorted. If anything, recent debates show that for the validity of the empirical findings to remain unchallenged rigorous experimental procedures and designs are mandatory. Yet, researchers involved in the current debate seem to disproportionately focus on outsmarting each other or proving each other wrong. We believe that this rapidly becomes counterproductive and leads to a stalemate in addressing the fundamental issues at stake. Therefore, we propose to organize a collective effort to systematically replicate the typological studies (e.g., perception of hills) across labs with the experimental protocols agreed upon beforehand (for a similar proposal, see Kahneman, 2012). Once the facts are clear, we can invest our thinking in new directions for a theory of embodied perception that unites affordance and spatial perception.
